# The challenges of the wind. Ecology of serious leisure in the Brazilian Northeast

**DOI:** 10.3389/fspor.2024.1406311

**Published:** 2024-08-21

**Authors:** Olivier Sirost, Bernard Andrieu, Jullya Bheatriz Dantas da Costa Sobral, Petrucia Da Nóbrega

**Affiliations:** ^1^ Sport Sciences Department, Univ Rouen Normandie, CETAPS, Rouen, France; ^2^ Sport Sciences Department, Université Paris Cité, I3SP, Paris, France; ^3^ Sport Sciences Department, Federal University of Rio Grande do Norte, UFRN, Natal, Brazil

**Keywords:** serious leisure perspective, wind leisure, wind challenges, Brazilian Northeast, body ecology

## Abstract

**Aim:**

The aim of this study is to understand the development of windsports and the challenges faced by wind-based leisure activities.

**Methods:**

The socio-historical dynamics behind the development of wind leisure are analysed on the basis of specialised tourism blogs, as well as a field study carried out in the Northeast Region of Brazil, combining ethnographic observations and semi-structured interviews with 6 wind leisure enthusiasts. All the data is processed using the serious leisure perspective tools developed by Stebbins, and the body ecology tools developed by Andrieu et al.

**Results:**

The results show that wind sports provide a dual continuum. First of all, there are the practitioners, from Neophyte to Devotee Worker. Then there is the junction between land, water and air, where wind practices ensure a dual aesthetic. On the one hand, riders are united by a community of flow, emotion and sensation mediated by their relationship with the wind and the creation of play materials (waves, lagoons, trade winds). On the other hand, the tourist development of these practices serves a political narrative based on postcard aesthetics, enabling wind concessions to be traded for the development of wind farms.

**Conclusion:**

this original research calls for a more systematic exploration of wind practices and the hybridisation of natural elements, which seems to illustrate the crisis that modern sport and its institutions are going through today.

## 1 Introduction

The objective of this article is to show and describe the development of winsport which finds a new space of expression in the Brazilian Nodeste without however being part of the institutional logic of modern competitive sport and Olympicism. Following the framework of reflection proposed by Serious Leisure, the article attempts to analyze how windsport is part of a set of sensory experiences allowing the incorporation of nature and its elements. The wind, beyond its ecological (renewable energies) and political (redistribution of populations and territories) issues, finds here a serious perspective in being part of a bodily ecology.

By analyzing the sensory matrices that windsport enthusiasts internalize and tell in their life stories, the article aims to show how an interior ecology of the living echoes an environmental ecology which is reminiscent of indigenous customs.

Wind challenges have become standardised sports and leisure events, newcomers to the culture of sensitivity to the weather (1, 2). Symbolically recognised in sail sports (3), these challenges, such as the Vendée Globe (“the wind of the globe”), or the Jules Verne Trophy (round-the-world sail race created in 1992) emphasise the confrontation with the deities of windpower. More recently, these challenges have been extended to the areas where sand and water meet. For example, the *wind challenge*, which is celebrating its 22nd anniversary in 2024, has been called the “biggest windsurfing competition in the world”^1^, bringing together some 1,200 riders in southern France, in Gruissan, a spot renowned for its strong gusts of the Tramontane wind. Described as the “Woodstock of surfing”, the event attracts both professionals and amateurs, “bringing together enthusiasts from 13 to 76 years old, in 3 disciplines: Windsurfing, Kiteboarding and Wingfoil”^2^. The activities also continue on the sand, with a village and dedicated festivities.

Here there is a mixture of the experiences of wind that marked the baby boomer windsurfers and their speed records, with acrobatic jumps and technical freestyle tricks. The main equipment manufacturers are based in the village on the sand. They give experts and amateurs an opportunity to talk to each other, as well as offering advice on equipment and equipment adjustments. Sails, foils, wings and boards designed for flying, surfing, waves, jumps and slaloms are all discussed with regard to honing, technical adjustments, adapting to the rides and spots, and the environmentally-friendly materials used in their manufacture. The place is animated late into the night with music, parties, drinks and barbecues. The spot, known for its powerful gusts of wind, is steeped in legend and the customs associated with the wind. J.P. Chassany (4) recalls the popular adage: “you recognise the weather by the wind, like the father by the child. Each windsurfing and wingsurfing spot is marked by its mythical winds, fertile or malevolent, whose symbolic practices leave their mark on the population and the land.”

J.P. Destand (5), in his wind ethnography of the Languedoc region, recalls how Mistral and Tramontane are incarnated in the bodies between land and sea in the form of living barometers. These can predict the formation of wet substances such as fog and waves, or solid substances such as sand whirlpools and sculptures in the seafront landscape. Weathervanes, road signs and warning signs, weathervanes on roofs, legends and old wives tales, fishing traditions and local wind products all reflect a sensitivity based on the feel of the wind, the perception of the changing colour of the sky and the water, and the variations in fragrances and scents that instruct the nose through movements in the air. Gruissan, a spot recognised by the Global Wingsports Association (GWA), is first and foremost an area bordered by marshes where salt is harvested at the Salin de Gruissan and its salt ecomuseum, by the Narbonnaise Regional Natural Park in the Mediterranean, the Sigean African Reserve (wildlife park) and the Sigean wind farm. Over and above the local traditions associated with the wind, the popular sports spot is also marked by its ecological dimension, encompassing concerns about preserving biodiversity, protecting sensitive natural areas and the use of renewable energy (6). A map marked by the wind is thus superimposed on an incarnation of windpower.

The sports equipment on display is also related to the disciplines and skills of windsports, sometimes with hybrids of sand, waves or water. Machines designed for speed, slalom, freeride, flight and waves, the equipment on display in the village provides an opportunity to talk about the wind and experience its manifestations. The language of windsurfing is spoken over the period of 4 days, conjuring up images of tug-of-war, the magic of runs, sailing in strong winds, the Tram (tramontane), wind direction, changes in the weather, the breeze and its wear and tear. While the village commemorates the rituals of hippie culture dedicated to sand and wind, with its village of tents and camper vans, refreshment stands and concerts, the practitioners are supported by real-time wind recording technology.

The Gruissan gatherings, as we will have understood, weave a link between the European communities (in Germany and Switzerland in particular) of nature spa visitors, those of the Californian cradle of the 1930s–1960s, and today wind sports tourism which invaded the Brazilian northeast. The preliminary analysis of the Gruissan festival makes it possible to provide a historical reading grid of the development of windsport in a social context marked by ecological policies, the safeguarding of heritage and traditions, and the economic issue of new forms of tourism (marked through sports practice and social media). It is important to enrich these observations with a literature review of these practices and their genesis.

## 2 Children of the wind: a literature review

At the turn of the 1960s, the combination of free time devoted to leisure and local and social sensitivities to the wind saw the emergence of new communities. Like the air-drinkers (7) and sun-seekers, a whole range of bodily practices emerged alongside the shift from traditional rural uses to leisure activities. Folklorists such as Van Gennep, and later sociologists such as Joffre Dumazedier (8), described the way nudism, camping, hunting and fishing, hiking and picnicking were among the many leisure activities that accompanied the rural exodus and the transformation of land areas, while at the same time maintaining beliefs and rituals in a natural temporality and pre-existing land areas (9).

The Languedoc region is no exception to this. The emptiness of the land (10), a space of perdition where wetlands and marshes reign, here remains the territory of the horse and bull breeders in the Camargue, the salt and reed producers, and small fishing villages. Some of these places were partially reinvested by a hippy counter-culture which, as in Beauduc, created a community of beachcombers (11) who lived self-sufficiently on the sandy lagoons. These new sand dwellers gradually imposed their seaside leisure culture, where recreational fishing, yachting and kite-surfing erased the former professional fishermen from the landscape. In the 2010s, these dreamers would find themselves expelled from their utopia by prefecture decrees in favour of nature conservation measures, under the cover of the *Languedoc-Roussillon Interministerial Coastal Development Agency*. In these superimposed layers of occupants, the native or local beach hut dwellers became undesirable, while the ecological lobbies led by the surfers’ associations imposed their political strength hold (12). These conflicts of use observed in France take place identically in the Brazilian northeast, as we will see later.

In the 18th century, kites became very popular in the Languedoc region, a way of depicting an animal cosmogony in the windy sky. They were to be re-purposed to be used in meteorology, sea rescue and pulling land and watercraft. In the 1980s, kite festivals sprang up all along the French coastline, including the international kite festival in Berck, the *Ronde des Vents* in Bray-Dunes, the Fréjus *Festival International de l’Air* and the Marseille *Fête du Vent*. In the Languedoc region, Narbonne plage and St Cyprien also have their own festivals. This rediscovery led to the emergence of new sports such as kite-surfing and parafoil sailing. Still in this transition from traditional activities to leisure pursuits, we should mention sand yachting, a swift means of transport favoured by the Chinese and the Dutch, which became a recreational activity in Belgium and France in 1898, and was promoted as an extreme expedition sport in the 1960s via crossings of the Mauritanian desert, giving impetus to new sports such as speed sailing and windsurfing. This detour through history shows the importance of reappropriating traditions in a context of development of sporting leisure activities. In the case of windsport here, traditions and technologies hybridize to form a new sensitive world.

It was surfers—particularly French surfers—who came up with the idea of transposing these techniques for capturing wind traction power from sand to water and waves. Brothers Joël and Arnaud de Rosnay were among the pioneers of surfing in Biarritz and Anglet back in 1957, forming the legendary group of “tontons surfeurs” (surfing uncles) (13). Joël de Rosnay, longboard promoter (who was introduced to the sport by the American writer and scriptwriter Peter Viertel) in the Basque country and France's first representative in international surfing competitions, introduced his younger brother Arnaud to the sport through the Waikiki surf club, an association he ran. Arnaud de Rosnay pushed the discipline towards extreme sports and the revival of the adventure sports in the 1970s. Thus, in 1977 he invented the speed sail—a hybrid object between a [https://fr.wikipedia.org/wiki/Planche_à_voile] and a [https://fr.wikipedia.org/wiki/Skateoard] and crossed a section of the Sahara in 12 days. For his crossing between the Marquesas and Tuamotu islands in 1980, he chose a kite moored to his board, known as a parafoil (the forerunner of kitesurfing). Always on the lookout for the sensations of wind power, he created the first windsurfing race in the Hawaiian archipelago: the Speed crossing. It was in this discipline that his partner, Jenna De Rosnay, was to excel and become the 1st ambassador of women's windsurfing. The way the brothers De Rosnay approached the practice is now considered as the promotion of eco-sports. This was treated theoretically multiple times in the scientific works of Joël De Rosnay (14–16), such as *Le Macroscope* in 1975, *L'Homme symbiotique* in 1995, and *Surfer la vie* in 2012, which emphasise the ecology of the living world. This philosophy also showcases practitioners and spokespersons who are directly involved in preserving the environment and wildlife (17).

**Figure 1:**
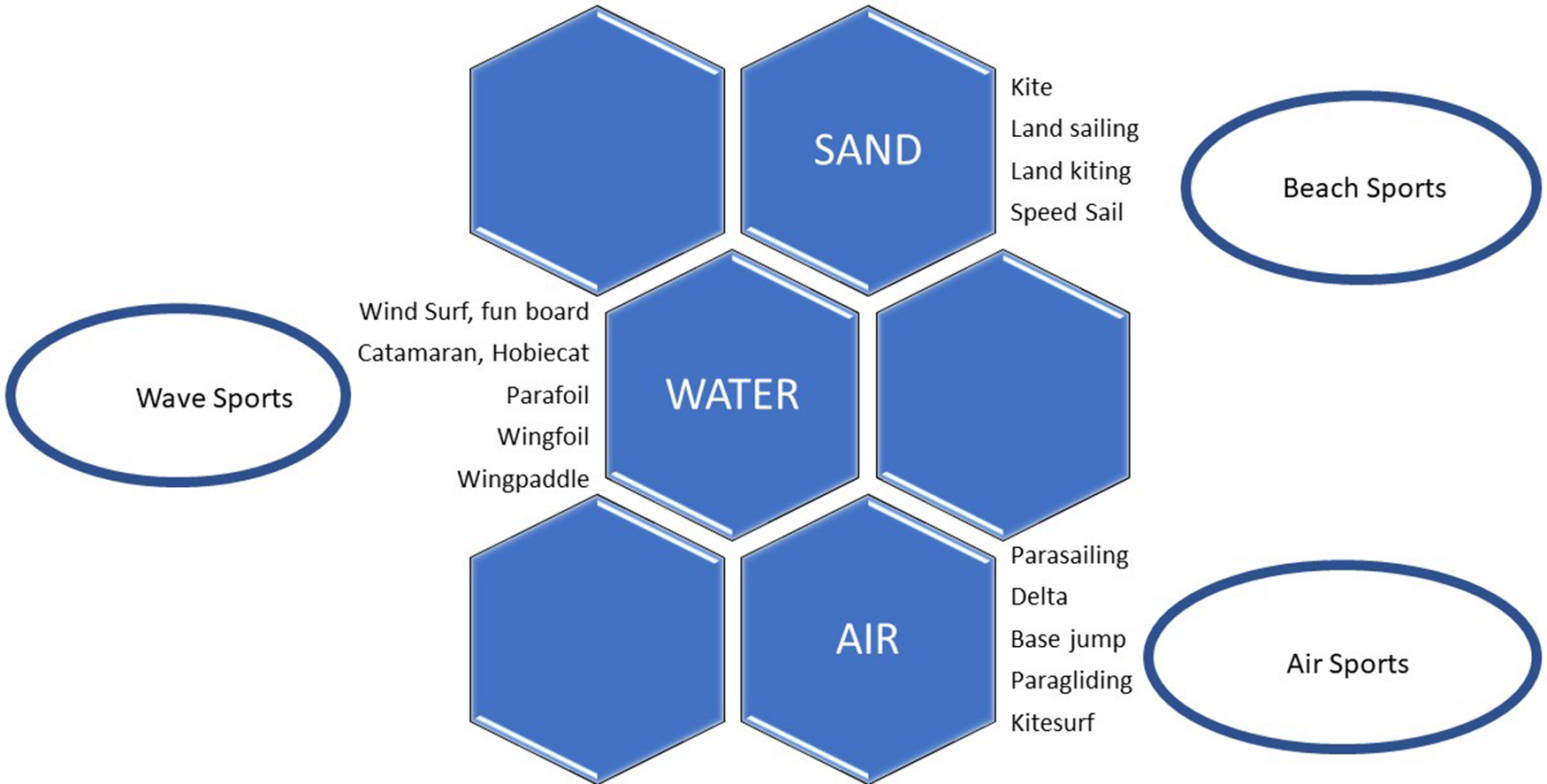
Below summarises all these wind practices. Windsports and their elements.

As part of this Franco-American dynamic, the children of the wind continued to perfect their technical objects. For instance, the windsurfer board was developed by aeronautical engineer Jim Drake in 1967 and pioneer Uli Stanciu; the first foils made their appearance in the 1980s, popularised by Éric Tabarly; the wind weapon was created in 1986 by Tom Magruber and Roland Le Bail; Tony Legooz's slingwing prototypes in 2011; the aerosail designed by Stéphane Rousson in 2014; and the adoption of inflatable wings by Robby Naish in 2019 (18). Wind messengers Kevin Langeree, Ruben Lenten and Kai Lenny followed in the footsteps of legends such as Kelly Slater, Daida Ruano, Björn Dunkerbeck, Eduardo Bellini and Sarah Quita Offringa, opening the doors to a wind tourism that migrated from Europe to South America. These historical elements can be synthesized in the relationships between sporting practices and natural elements (Figure 1).

In addition to the regattas approved by the International Olympics Committee (IOC) (19) and the assembly of windsurfing professionals and equipment manufacturers through the Global Wingsports Association (GWA), most windsurfing spread via the social media of tourism that are flourishing on the internet and turning these winged deities of windsport into influencers. Former engineer Isabelle Fabre admits she gave up everything to live a life of adventure and travel, learning to kite-surf and wind-surf as she travelled the world with her drone in search of the wind^3^. Now a “digital nomad”, she organises camps and trips for wind seekers. *Glissevolution*^4^ offers online travel guides for kitesurfing enthusiasts, as well as information on spots, accommodation and equipment hire. *Spots Evasions* is a tour operator specialising in “thrill-seeking trips”^5^, which has been in business since 1996 and offers turnkey experiences for beginners through to advanced practitioners. Wingfoil, wingsurf and winsurf are all part of a wide range of wind sports. The Brazilian Northeast is featured in this tourism offer for specialists, adventurers, nature lovers… as a fashionable destination, allowing the challenges of the wind to be considered from a serious leisure perspective.

## 3 Wind challenges in serious leisure perspective

If we examine the way population groups seize these wind practices, we can show, from the serious leisure perspective developed by R.A. Stebbins (20), that there is indeed a continuum around wind challenges. This continuum goes beyond the institutional dimension of sport and its goals of competition and performance. Nor, as Stebbins shows (20), can it be explained simply by reducing it to the aspect of risk-taking, confrontation with extremes, adventurousness or an alternative lifestyle. To overcome this impasse, we need to examine the reciprocal relationship between playful involvement and the natural environment, in this case the wind. This intuition was confirmed by R. Melo et al. (21), who show how, over the past few decades, Nature sports activities have unleashed an avalanche of new paradigms to characterise sport and its new educational, health, environmental, tourism and hedonistic challenges. According to Davidson and Stebbins (22) nature-based leisure activities can be understood from the perspective of sustainable development by examining the reciprocal relationships between natural elements and bodily practices.

However, the analysis comes to grief against a very Western classification of the natural elements (with reference to the correspondences between elements, passions and temperaments from ancient Greece as analyzed by (23), forcing the opening of a final chapter on hybrid activities. Unsurprisingly, wind practices fall into this category, escaping the categorical confines of air, water, earth or snow and ice: “Indeed certain activities can be considered hybrid NCAs (nature challenge activities), as they involve simultaneous engagement with more than one natural element. These include the wind-propelled activities undertaken on water, land, ice or snow” (Davidson & Stebbins, p 179). The fluid transition between elements offered by practices such as kitesurfing and windsurfing opens up the potential for action that involves livelihood, contemplation, movement, play, thrill seeking or a seemingly limitless optimisation of sporting performance. Adventure, career volunteering are the forms of leisure that best combine all these latent potentialities, and make it possible to go beyond the non-homogeneous and sometimes incoherent sporting classifications proposed by sports federations. Recent literature on the practices emphasises these dimensions. The development of guides, indexes and bio-meteorological tools that comes along with the proliferation of environmental & weather challenges in outdoor sports (24) is a remarkable feature of wind leisure. Specialists (25, 26) stress the relationship between what is carried by the wind (temperature, dry humidity, debris, climatic accidents) and the sensory organs (in particular the skin), and training in meteorology (27).

**Figure 2:**
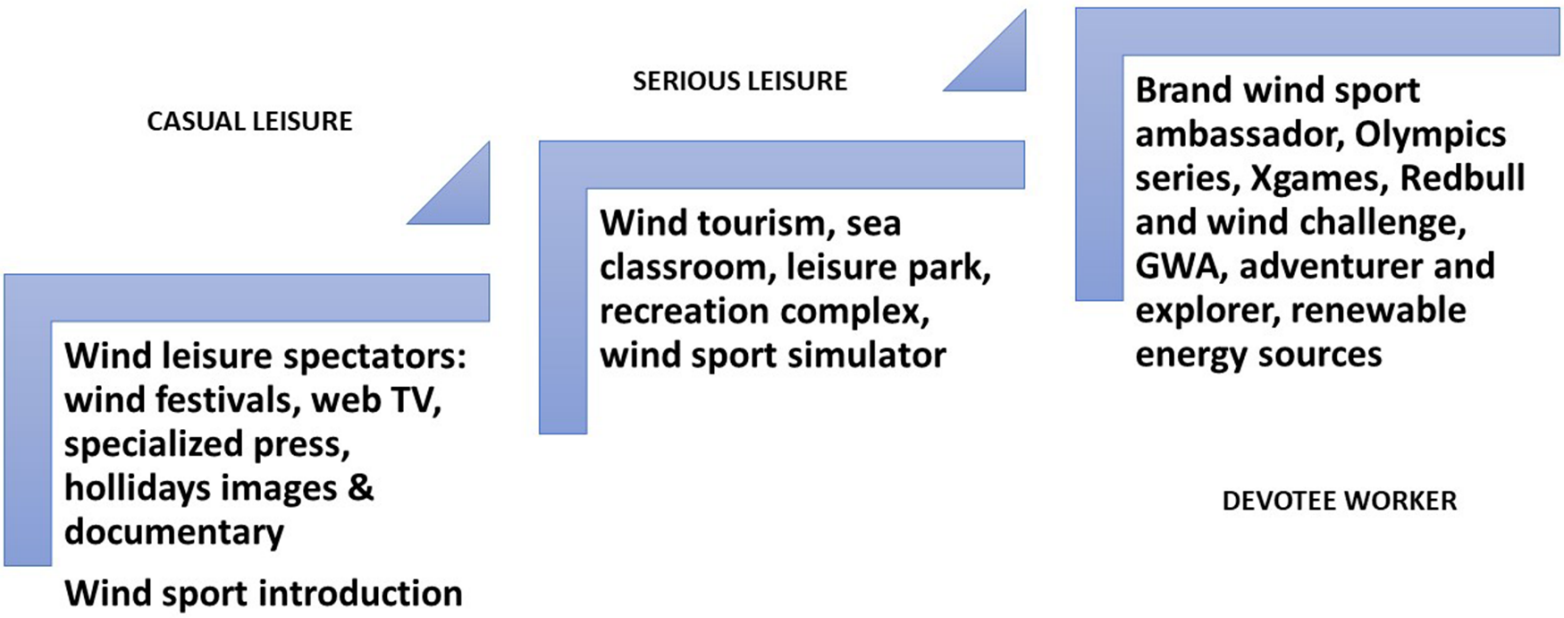
Serious wind leisure perspective: involvement scale.

If we transpose these lines of thought onto wind leisure [as (28) did], this allows us to uncover the following scale (Figure 2):

Wind casual leisure refers to the development of alternative media and festivals, which quickly grew in the 1980s with the Nuits de la Glisse (short film festivals), wind festivals (kites, then windsurfing and wingsurfing), media dedicated to these practices, weekend offers (such as smartbox or wonderbox) and holidays (29), as well as first flights, skydives and other immersive experiences in the wind.

Wind serious leisure is embodied by educational offers to raise awareness of the environment, combining sports activities and meteorological knowledge; but also by the proliferation of leisure parks and centres, or even recreational simulators to experience the sensations of wind power (wind tunnel mechanisms, generating currents, for example). Above all, it is the planetary-wide development of wind tourism that should be highlighted, enabling the cosmos of air, water and sand to be brought together, while promoting a dream landscape of sensation in which it is pleasant to let oneself be carried away (30).

Finally, a whole host of wind devotee workers have come to the fore, renewing the pantheon of winged sporting heroes, softening the image of wind entrepreneur, brand ambassador, wind explorer or Alizée challenger (such as the Red Bull King of the Air).

And so the interpretation grid of wind-related practices comprises being part of the landscape, the use of meteorological tools, the flow of sensations, and ecological awareness (protecting the environment). Roger Caillois once classified these activities as vertigo games, the aim of which was to destabilise the senses and perception (31). According to Bernard Jeu, such practices are part of an ordeal in which the pursuit of the exceptional feat, risk-taking and the fantasy of the environment provide access to the realm of the marvellous and the extraordinary. From primitive tribal initiation to professional vocation, the ordeal sketches a confrontation with the natural elements, a technical incorporation of matter, and a plunge into the depths of the self (32). For the ethnologist Christian Bromberger (33), rather than leisure activities, it would be more appropriate to talk about passionate people, that he proposes to divide into five categories: tinkerers, adventurers, seekers of knowledge, participants in shows and games, and seekers of meaning. The psychological state of ordinary devotees and their attachment to the object of desire reflects what the ancients called the agitations of the soul. This perspective is taken up by sociologists J. Griffet and C. Martha (34) in their analysis of base-jumping, which combines the use of social media, technical equipment for flying and falling, awareness of the risk of a fatality, the search for a state of flow and the pleasure of toying with limits. This work is in line with the perspectives developed by M. Zuckerman on the quest for thrills and sensations in extreme sports (35), or the non-rational overflowing of emotions, sensations and images, which are particularly present in sports involving real danger, as described by M. Apter (36). Here they are rooted in the risk-taking associated with the perception of the wind (37) or emotional stress (38). Sam Elkington (39) emphasises the importance of flow in understanding the development of these ways of practising nature and their vocation to produce aesthetics. The experience of flow envelops the practitioner in a halo of intense sensations and emotions, producing a feeling of being outside oneself and one's surroundings (short-circuiting space and time), a feeling of harmony and being at one with the landscape. In this respect, the experience of flow reveals an ecology of optimal leisure.

## 4 Body ecology to understand wind serious leisure

For our part, we have developed the hypothesis of a body ecology characteristic of the renewal of leisure practices (40). These practises are based on the logic of emersion, immersion and inhabitation, which replace the modern sports motifs of competition, performance and breaking records. The somatic practices of yoga, reflexology and mindfulness meditation that are associated with the surfing lifestyle are the result of an emersion of the sensations of the living body, which becomes a sought-after goal. Activities involving immersion in nature, such as walking, trekking or hiking across rocky massifs or sand dunes to reach the seaside, encourage the rediscovery of oneself through confrontation with a natural environment and also become a goal to be pursued. Finally, activities such as camping (41) or nudism that involve living in nature help to build eco-soma-systems, or living landscapes of leisure.

Body ecology is a micro-ecology: “It is because we transform the practices of individuals that we transform the ecology of the world. The idea is to experiment with changes in sensory practices. To experience our environment differently, we have to be willing to disorientate ourselves in order to allow other modes of existence, movement and relationship to emerge” [(40), 12]. According to Andrieu (42), immersing oneself in or gliding over water “activates new resources of the body by plunging it less into vertigo or risk than into ecological immersion” [(42), p. 131; 132]. This understanding was confirmed by the windsurfers, kitesurfers and stand-up paddleboarders we interviewed on the beach at São Miguel do Gostoso. These board sports combine water and wind, but also sunshine. They are characterised by immersion in the movement of nature, involving “a bodily commitment that extends to being enveloped by the fluid element” [(42), p. 134].

Through its capacity to adapt in the very course of its ecologisation in the air, the living being of our body anticipates without the consciousness of the lived body being able to control it. A succession of experiences in the air can reduce this involuntary activation of the living body, but it never disappears despite hours of training. The search for new spots with different climate conditions (wind, exposure, relief, height) fuels the desire to renew the body schema through pre-motor activation.

This sharing of an immersive life in a slower time (43) enables us to savour encounters and learn another way of living that is closer to our feelings and emotions. But it also requires us to broaden our perception and our solidarity so that we can have real exchanges with the local population, for example to deepen our listening skills when we were talking to the locals and, little by little, discover their way of life, the social difficulties of the region, their wishes, etc.

### 4.1 Methodology

**Figure 3:**
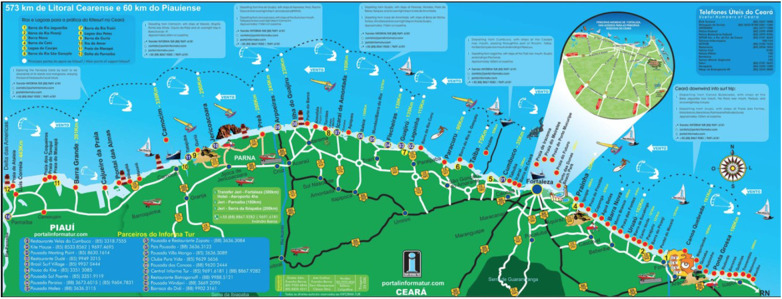
Map of kitesurfing and windsport spots in the Brazilian northeast. Source: http://kiteandsmile.blogspot.com/2016/08/kitesurf-au-bresil.html.

In recent years, Brazil has become the new Mecca of winsports. Tour operators promote destinations with heavenly and pristine natural landscapes. The accommodations reflect an ideal of free living marked by the hippie culture of the 1960s, an abundance of wings, sails, sand and waves; but also a continuous wind and spots marked by wind power or clemency. The map below reflects this postcard image quite well (Figure 3).

The aim of this article is to present sports tourism in São Miguel do Gostoso from the point of view of body ecology (44) based on first-person accounts from water sports enthusiasts in the form of interviews with professional and amateur athletes. Between April 20 and 22, 2018, we interviewed twelve practitioners aged between 17 and 45. Among them were tourists, owners of windsurfing and kitesurfing clubs, instructors, athletes including Kauli Seadi, the 2005, 2007 and 2008 world windsurfing champion who runs the club and the Kauli Seadi Pousada in São Miguel do Gostoso, where we stayed.

The results of our fieldwork are based on six interviews with sports fans during our research trip. We stayed at Club Kaouli, where we paid for our stay and were able to take stock of the club's activities. An analysis of the qualitative interviews with the curriculums reveals three kinds of relationship with bodily ecology: on the one hand, **an ecology of the element,** in this case water and wind, with the founder of the place Kauli Seadi by the conversion to tourism of the world champion Brazilian wind-surfing waterman. Secondly, **a sensory ecology** through the experience of immersion in the element to discover the intensity of internal sensations. Thirdly, a **sustainable ecology** that places local players in a sustainable health economy, taking advantage of the changing winds to redefine their place:

Although they often work in the same wind tourism networks, each of the groups studied are in a different position and have differing economic resources for developing their relationship with the elements.

São Miguel do Gostoso near the city of Natal in the state of Rio Grande do Norte is a new windsurfing destination, offering the same good conditions with four beaches, flat water, protected by reefs and waves of about one meter. The wind is strongest from September to March: from 15 to 30 knots.

New now in addition to the Kauli bungalows, discover the “Village Kaudi Seadi” 150 m from the center and the beach.: “You want to stay with friends or family, feel at home, the Village Kaudi Seadi offers apartments with a living room with tv sofa, a fully equipped kitchen, 2 bedrooms and a bathroom. Balcony with hammock, garden view”The Clube Kauli Seadi is set on Cardeiro beach just outside the village of Sao Miguel Do Gostoso. The centre offers a great base to enjoy the different sailing areas for all levels—particularly wave sailors. The area has consistent trade winds that blow pretty steadily from August to March. At the start of the season the wind is offshore and changes direction from October to March to a side shore wind. The wind is strongest from August - November (usually around 20–30 knots), whereas from December to March, the wind speed average is 15–25 knots. The centre is located at the base of the lagoon with flat waters providing safe and easy learning conditions.

The interviews took place at the Club Kaouli itself during a series of stays there as part of the research internship of one of the authors. But in order to have a separate point of view, we insisted that each person's curriculum distinguish different levels of experience. But with interviews with people of different status in the organization, we were able to reconstruct the network.

These 6 participants distinguish the champion creator of the tourism company Kauli and the different levels of practice and gender. The relationship of the participants in the study is not only the location of their practice in the same club but also the degrees of sensitivity to nature, the element wind and the uses of their body in the practice. Their functions in local tourism are also clearly identified in the service of the overall network.

In the interviews we favored information relating to sensory data, as suggested by the work carried out by David Howes (45). In the first-person account given by the interviewees, we noted in the verbatims how the emotions and sensations linked to the wind explain the relationship to sports practice, the territory and tourism.

Sensory phenomenology is privileged here by a first-person perspective (46). The view from within: First-person approaches to the study of consciousness. Imprint Academic.): because it is the sensory impressions of the wind that constitute a perception collected in the verbatim of the interviews. The sensory impression thus serves as a guide to appreciate the degrees of body ecology (40) particularly in the cosmic contact with the element of wind, here.

Different degrees of wind sensations are described here, ranging from simple atmospheric aeration to immersion in the elements: thus Kauli tells us that beyond the adrenalin of sailing, Silvio describes the sensation of freedom “You feel free. It's the best way to feel nature”, Ana Paula through the relaxation in the element feels in a different state of consciousness, Clenilson specifies the degree of immersion into the element.

### 4.2 The Brazilian Northeast: a land of wind

Today, the challenges of the wind are being met in the Brazilian Northeast (47), with popular destinations for wind surfing, kitesurfing and wingsurfing. Depending on the destination, these activities are complemented by sailing, fishing, stand-up paddling, hiking, buggy rides, surfing in the waves and on the sand, beach volleyball, diving and snorkelling (44).

The commercial tourism websites^6^ provide a map of windsport spots ranked according to waves and bodies of water, ease/difficulty of access, wind intensity and variability, landscape composition (dunes, water, lagoons, characteristics of the sand or rock, vegetation, animal life), climate and meteorology, which are the main indicators for creating a continuum between Neophyte and Devotee Worker. Each spot is described and illustrated in terms of the landscape, the services and accommodation available, access to windsporting equipment, the history of the area and the biodiversity present, all of which connect the spot to the level of practice. Alternating between travel guides, holiday sales pitches, accounts of experiences and advice from sportsmen and women, blogs and websites present the village of Jericoacoara as a world reference for kitesurfing, the village of Cumbuco as the stronghold of professional kiters, and the Brazilian village of Barra Grande (which hosted the freestyle kitesurfing world championship in 2014) as the ideal place for beginners, with strong winds of up to 35 knots and shallow water at low tide (48).

The Brazilian Northeast has undergone major changes in recent years as a result of policies to govern nature (49). Two main factors underpin this regional modification: the development of tourism and the presence of environmental institutions. Conceived as part of a policy of common good, nature has seen environmental bureaucracy take over from customary management. The historical arrangements between indigenous natives, farmers and the relegated members of society (fishermen, hippies) have been undone by the policing of environmental behaviour. This disciplining of nature has been achieved in particular by using tourism as a watchtower for ecological behaviour deemed to be deviant, and by marketing wind concessions to produce wind energy (50). Laloum's ethnographic study of Praia da Pipa shows how wind tourism, developed by surfers and travellers from the urban and counter-cultural bourgeoisie of the 1970s, gave impetus to measures to protect the flora and fauna, while at the same time overturning cultural traditions. The fishermen's huts, which used to have their backs to the sea to protect them from the wind, have become tourist bungalows with a privileged view of the practice spots. Environmental education for local populations has also become a social issue. The traditional ecological wisdom of the peoples of the wind living in the primal forest—such as the Minuano Amerindians and the Guaraní people—had built a cosmology of the wind around the figure of Namandu, the original fertilising wind. In the same way, farmers and fishermen had learned to cultivate and garden nature according to the knowledge transmitted by the Alizées (43).

For commercial reasons, wind tourism is being used as an alibi for a property boom in which the view of the landscape becomes a postcard scene presenting the ideal spot for riders (51). The situation for coastal leisure activities appears to be similar for mountain sports in Brazil (52). In this instance, the aesthesis of serious leisure confronts the frozen aesthetics of the postcard and its morality.

In the face of these excesses, the ecological economy of a sustainable market is currently at stake in the village of São Miguel do Gostoso: in trying to give priority to local players, the tourist board is having to contend with German offers to develop the real estate on offer: “The Chairman, Managing Director, Representative, Senior Advisor, Marcelo Queiroz and the Coordinator of Cable Development Projects and Industries Trier (EIC Trier), Matthias Fuchs, Project Director and Head of the Escola Senac Barreira Roxa Hotel, are planning to amend the project. A new conference with the German industrialist has been set up for the period from 2019 to 2021, with no dialogue on nature protection in place. It is important to know whether our compromises are improved as such, as described in the statistics, by the voice of the government, through the social and economic decision form, by Marcelo Queiroz.”^7^

The arrival of the European tourists, ready to invest, is in stark contrast with the small shops working from inside the houses. Tourism for the locals includes both traditional handicrafts such as items made from coconut and baskets woven from palm leaves, and industrial products that are beginning to colonise the stalls, with a shop selling Havaianas flip-flops in 2018 as the start of a tourist showcase for a clientele that is more seaside than local. The contrast between the self-sufficient economy of the fishing village and the expansion of the seaside resort market is reflected in the resources available for leisure activities.

The cartographic approach, no longer merely quantitative and touristic, becomes a topophilia (53) which establishes, according to a humanist model of geography, the affective link between a place and a practice. Local variations in sporting practices, their spatial distribution patterns and socio-spatial studies of sport reveal the extent to which place and space affect the way people experience their bodies. Land-use planning for physical recreation does not serve the rationale of sensory immersion in nature. The feeling of space is created by contact with the earth and the elements it produces in a given area.

A new form of eco-tourism for visiting these treasures of the Sertao^8^ is now developing from the rationale of development. Eco-tourism is therefore a practice that is in the process of developing, with, so far, no education on nature being offered. The great outdoors and adventure (54) in a sports version has already opened the way for the general public. Environmental education by the Brazilian State was the forerunner to ecotourism from 2005 onwards^9^.

Paulo Freire (55, 56) in The Pedagogy of the Oppressed opened a new path in the 1970s, showing how practical and concrete means allow true conscious self-liberation. It is not a question of a simple reversal of roles between oppressors (here the State and the banks) and oppressed (here natives and fishermen), but of developing an ecology of human relations based on the sharing of knowledge, traditions and philanthropy. Faced with the common good that wind represents in the Brazilian northeast, we can bring Freire's intuition back to the practical role played by windsports and their diffusion in the face of a State which markets wind concessions for wind turbines with complete impunity.

The challenge is not only to raise awareness of our geological heritage, but to open up a cosmic sensibility. The place becomes inhabited by myths of healers communicating with telluric energy (57).

## 5 Portraits and testimonials

### 5.1 The place for wind tourism

Kauli Sedi has built a beach club to welcome visitors and organise surfing courses on site in a windy location.

São Miguel do Gostoso is also a place where ecological ideas are disseminated (58) through the *Gostoso* Film Festival organised by Heco Produções and the Collective for Human Rights, Ecology, Culture and Citizenship (CDHEC). As well as daily screenings of the films and discussions with the producers on environmental transformation processes, the festival offers a series of technical and audio-visual training courses for over 50 young people from the municipality and surrounding districts. Since 2013, this group of young people has organised over 30 workshops and produced ten short films. The project has become a major event in the national film scene and one of the most important in the state of Rio Grande do Norte.

São Miguel is a small fishing village 100 km from Natal. According to the IBGE (Brazilian Institute of Geography and Statistics), the population in 2017 was around 9,600. The region's economy is centred on fishing, tourism and wind power. Since the 2000s, São Miguel has become a tourist destination mainly for water sports, gastronomy and the film festival. It is also a place for those seeking peace, relaxation and meditation by the ocean (47).

The village of São Miguel de Gostoso and its beaches has become an international tourist destination, including for sports tourism. On the “Spots d'Evasion” website, for example, you can find information about “windsurfing holidays”: “Sao Miguel, the destination for your windsurfing holiday in Brazil, 120 kilometres north of Natal, has a number of immense bays partially protected by sandbanks, where the waves roll out warmed by the sun. The palm trees lean over to enjoy the spectacle of the wings and sails of the windsurfs gliding over the waves. The village fishermen never miss an opportunity to surf the best waves, ensconced in the back of their Jangada”^10^.

### 5.2 Windsurf in São Miguel do Gostoso

Kauli Seadi was born on 20 December 1982 in Florianópolis. He started windsurfing at the age of 12 and at 16 became a professional athlete, winning the first professional event of his career at the age of 18. He is an 11-time Brazilian windsurfing champion and the 2010 Brazilian stand-up paddle champion. Brazilian Kauli Seadi is an icon of the sport: he invented manoeuvres, innovated in the way he navigated the waves and developed new models of boards. On his website^11^ you can find out more about his sporting career and his club, which focuses on tourism for sports and well-being enthusiasts.

He currently hosts the Waterman programme on the OFF channel with his wife, oceanographer Nana Seadi. The *Kauli Seadi Club* in São Miguel do Gostoso is a reference when it comes to equipment hire and lessons for windsurfing, kite surfing, surfing, tennis and stand-up paddling. His passion is being on the water, whatever the sport: windsurfing, kite surfing or stand up paddle. Every year, especially during the Brazilian summer, he welcomes tourists, mainly Europeans, who come to São Miguel to practise their sport.

The *Amjus* Association (Association for the Environment, Culture and Social Justice) is a non-governmental organisation created from the feeling of a group of young students and professionals from the community of São Miguel do Gostoso who were already involved in social commitment through other actions and projects, and wanted to make a social commitment by founding this NGO in January 2009, including as a research institution. The main activities of AMJUS are research and environmental education, focused on the conservation of sea turtles in São Miguel do Gostoso, combining educational activities with projects for the social and human development of children, adolescents and young people in the community. Club Kauli Seadi is one of the NGO's partnerships.

As a tourism entrepreneur, Kauli Seadi tells us that he has been well received in the village and that there is a consensus that development is good for everyone working in the area, not just for the few. During our stay at the club, we noticed the good relations between him, the staff and the local population. Sailing sports are expensive and reserved for an upper-middle class population. In São Miguel do Gostoso, some clubs are starting to make these sports more accessible not only to tourists, but also locally by investing in social projects. From this perspective, in his club he is fostering a social education project, *the Wind project,* which aims to teach windsurfing to children and young people in the town. To take part, they must attend school and achieve good grades.

During the interview, Kauli Seadi tells us that through sport he has travelled a lot, learning about other cultures and ways of life. For the past seven years he has chosen to live in São Miguel do Gostoso because there is excellent sailing every day, friendly people, sunshine, warmth, beaches and lagoons to enjoy different water sports. We stayed for three days at Club Kauli Seadi to observe, to establish the research area and to enjoy the atmosphere, which was conducive to body ecology and beach leisure, surrounded by sun, warm sea water, wind and sand.

Kauli tells us that beyond the adrenalin of sailing, he takes the opportunity to relax and find balance in his life, and to be closer to his family: he is married and the couple are expecting their first child, a little girl. When asked about the risks of sport, he said that in dealing with Nature he is dealing with an unpredictable element, so at the same time he strives to respect the conditions of the environment and challenge the elements to overcome them. He admits that when he began, he was scared, but that he overcame his fear through technique and gained in confidence.

Silvio Vilarine, 28, is currently a DJ, but before that he was a professional *kitesurfer*. He has taken part in several national and international circuits throughout Europe. He now enjoys sport as a hobby: “It's one of the best sports I've ever done in my life, so I want to do it until the day I die. It's an indescribable sensation—you have to be in the water to experience it. Sliding into the sea, jumping. You feel free. It's the best way to feel nature, because you're with the wind and the water”.

He tells us that, of course, to be able to take advantage of this, you need to have the technique, because there are risks and dangers involved. You need body technique, and there are several courses in the region to teach this. He says that Rio Grande do Norte has a lot of potential for the development of sport and sports tourism. But there is an even greater lack of funding and projects to develop education and the environment.

Ana Paula, 43, lives in Rio de Janeiro and was in São Miguel on holiday. She tells us she loves water sports, especially windsurfing, which she has been doing for a long time, and more recently *kitesurfing*. Her love of the sea and sailing sports comes from her family, from her father, Ana Paula confides with emotion. She tells us that when she's sailing, she has a feeling of pleasure and wholeness. We can characterize these interviews into three main types of ecologies (See Table 1).

**Table 1:** Cross-referencing of the 3 types of speech analysed.

Types of body ecology	Types of tourism	Sensation	Sport	Agency	Social development	Structure
Ecology of the element	Sports tourism	Balance	Wind surf ki-paddle windsurfing	Tourist entrepreneur	Environment, culture and social justice association	Club Kaoli Pousada
Sensory ecology	Immersive tourism	Pleasure and wholeness	Sea and wind immersion	Kite surf instructor	Tourism training	Sports activities
Sustainable ecology	Sustainable tourism	Well-being	Reception of sports tourists	Local developer	Native cultures	Windsurf School (Dr. Wind) and inn

She notes that in the sea there is no repetition, unlike in a swimming pool, thanks to “the movement of the tide, the direction of the wind, the intensity, the landscape”. She spends several hours in the water, all afternoon, until sunset, feeling touched by the grandeur of nature and the contact with the elements. She tells us that she feels stronger when she's in the sea: “It's my home, I feel safe and more physically receptive… I feel I am in a state of relaxation“. She tells us that after practising sport she feels in a different state of consciousness. This sentiment is shared by other practitioners we spoke to.

Bruno de Sant'Anna was born in São Paulo, is 34 years old and has been a kite surfing instructor at Club Kauli Seadi for three years. He tells us that he fell in love with the region mainly because of the opportunity to practise sport and also to be closer to the sea, so he decided to stay. He already had professional experience, having taken several training courses, including in Europe, to teach kite surfing. He notes that sport brings him into contact with nature: “It is a sport that combines two forces of nature, two natural elements, water and wind. We are connected to these forces. When we are out sailing, we see fish, turtles and dolphins. For me, that's quality of life”. He goes on: “When I'm far out [to sea] the only sound is the wind and the water. I feel the wind in my ears, I feel gratitude for living in this moment, so I have a feeling of wholeness. I don't really know how to explain it. It's the relationship with nature, it is very moving emotionally, so it's difficult to explain in words”. Other practitioners we interviewed also spoke of the sensations of relaxation, a feeling of gratitude and sharing with nature, as well as a greater awareness of the environment (43).

Clenilson, nicknamed *Rolinha,* age 17, was born in São Miguel do Gostoso. He has been involved with Project Wind at Kauli Seadi for two years. He confides in us that he literally feels like a fish in water. He tells us that his friend Rubinho has now moved to France to work and train in windsurfing. He is well aware of the importance of this project for the children and young people in his community, as a way of looking to the future and educating them through sport.

## 6 Conclusion: the two directions of the wind

This exploratory work ultimately shows that faced with the Brazilian State and the green wind economy, windsport tourism represents a serious alternative. It allows us to reestablish a link between fishermen and waterfront recluses, natives and farmers, sports enthusiasts and local populations. The teaching of windsports and their appropriation by Brazilians plays the card of a bodily ecology where the multiple experiences of the wind connect the different actors of the Nordeste. Thus, the practices of windsurfing, kitesurfing and wingfoil are not a simple tourist economy, but real practical acts of resistance to political power and energy issues. Faced with the green colonialism of wind turbines, as Freire indicated, a pedagogy of the oppressed is being put in place, which aims to be a liberating internal revolution. Windsports, as evidenced by our interviewees representing these new wind players, allow us to reconnect and reconnect with our native soil and to have this cultural originality recognized throughout the world via tourism and sports gatherings.

At the end of this investigation we see that wind leisure constructs a double discourse. On the one hand that of the win-win renewal of Brazil's national economy and the preservation of biodiversity through the challenges of wind (59). On the other hand, that of police of ecological behavior which crushes the myths and ancestral traditions of the wind carried by the natives and the farmers.

On completion of this survey, we can see that the wind leisure companies are constructing a double narrative. On the one hand, there is the win-win renewal of Brazil’s national economy and the preservation of biodiversity through the challenges of wind (59). On the other hand, there is the policing of ecological behaviour, which crushes the myths and ancestral traditions of wind that thrive among the indigenous peoples and farmers.

Testimonies about wind sports show that, far from the images fixed by the dream of the birdman and the idyllic landscapes of holiday promoters, wingfoil, windsurfing, kitesurfing and other related sports bear witness to a shift in patterns and experiences of physical leisure activities.

For a long time, sport and the leisure time hard-won from working hours were seen as a showcase for progress and modernity. Here we see that it is not the metric or chronometric performance, or the setting of an absolute record that is sought, but the flow, the feeling of being an integral part of the landscape or witnessing the birth of a somatic cosmos.

Could we not say that wind leisure may be a sign of a new era—despite attempts at political and economic recuperation—and a return to a hermetic tradition? By combining the contributions of the serious leisure perspective and body ecology, this study is an invitation for others in the same vein.

## Data Availability

The original contributions presented in the study are included in the article/Supplementary Material, further inquiries can be directed to the corresponding author.

## Footnotes

^1^ https://www.windmag.com/actu-defi-wind-2022-tout-monde-retient-souffle-sauf-vent

^2^ https://actu.fr/occitanie/gruissan_11170/du-mondial-du-vent-au-woodstock-de-la-glisse-l-aude-releve-tous-les-defis-sur-l-eau_58785367.html

^3^ https://isabellefabre.fr/quisuisje/

^4^ https://www.magasin-glissevolution.com/blog/guide-kite-trip-dans-le-nordeste-bresilien-n645

^5^ https://spots-evasion.com/region-pays/bresil/

^6^Worthy of note: spots-evasion.com; brazilecotour.com; /windsurf.voyages-adekua.fr; fun-and-fly.com; magasin-glissevolution.com; isabellefabre.fr; terra-nordeste.com.

^7^http://fecomerciorn.com.br/noticias/premiado-pelo-mtur-projeto-verena-do-sistema-fecomercio-rn-em-parceria-com-a-alemanha-e-renovado-ate-2021/ Accessed on 23 June 2019

^8^http://revistaecoturismo.com.br/turismo-sustentabilidade/nordeste-imperdivel-conheca-cinco-destinos-paradisiacos-da-regiao/ http://www.penaestradatrilhas.com

^9^MINISTÉRIO DO MEIO AMBIENTE. MINISTÉRIO DA EDUCAÇÃO. *Programa nacional de educação ambiental - ProNEA.* 3. ed. Brasília: Ministério do Meio Ambiente, 2005.

^10^https://spots-evasion.com/sejours/sejour-windsurf-a-sao-miguel-do-gostoso/ Accessed 21 June 2019.

^11^ http://clubekauliseadi.com/
